# Application of oral fluid for quantitative nucleic acid and antibody detection of the severe acute respiratory syndrome coronavirus 2

**DOI:** 10.3389/fpubh.2025.1659411

**Published:** 2025-12-03

**Authors:** Rui Song, Jun Li, Yiting Wang, Qi Huang, Yan Geng, Xiaoyan Ding, Xuecong Duan, Chunli Wen, Yanfen Tang, Lu Kang, Fang Huang, Mei Dong, Jianping Dong, Xiaoyou Chen

**Affiliations:** 1 Beijing Ditan Hospital, Capital Medical University, Beijing, China; 2 Beijing Haidian Hospital, Beijing, China; 3 Beijing Center for Disease Prevention and Control, Beijing Research Center for Respiratory Infectious Diseases, Beijing, China; 4 Beijing Research Center for Respiratory Infectious Diseases, Beijing, China

**Keywords:** oral fluid, SARS-CoV-2, dPCR, RT-PCR, IgG

## Abstract

**Introduction:**

To evaluate the performance of oral fluid (OF) for quantitative detection of SARS-CoV-2 nucleic acid and IgG antibody, and explore its application value, we compared OF with traditional samples using multiple detection methods.

**Methods:**

Real-time PCR (RT-PCR) and digital PCR (dPCR) were used to detect SARS-CoV-2 nucleic acid in 213 paired OF and throat swab samples from COVID-19 cases. Chemiluminescence was applied to detect IgG antibody in paired OF and serum samples, while colloidal gold method was used specifically for OF.

**Results:**

RT-PCR results showed that the positivity rates of OF and throat swabs were 80.75% (172/213) and 88.73% (189/213) respectively (*p* < 0.001). Using throat swabs as the reference, the sensitivity of OF for nucleic acid detection by RT-PCR was 87.83% (172/189). For 108 paired samples analyzed by dPCR, the positivity rate of OF was 86.11% (93/108), slightly higher than that by RT-PCR (85.19%, 92/108). Notably, in some cases, the viral load in OF exceeded that in throat swabs, accounting for 38.89% (N gene) to 41.67% (ORF1ab gene) of the tested samples.

**Conclusion:**

As a non-invasive, convenient, safe, and self-collectible biological sample, OF shows high consistency in detection efficacy compared with traditional throat swabs and serum. It thus holds important application value for the diagnosis, monitoring, and epidemic prevention and control of COVID-19.

## 1 Introduction

The coronavirus disease 2019 (COVID-19), caused by severe acute respiratory syndrome coronavirus 2 (SARS-CoV-2), has created a considerable disease burden and public health threat worldwide (1, 2). Moreover, large-scale and long-term epidemic surveillance poses new challenges to existing sampling methods. The process of large-scale throat swab sampling is time-consuming and labor-intensive, and the risk of cross-infection brings significant physical and mental stress to the sampling personnel. Therefore, there is an urgent need to develop an efficient, safe, convenient, and self-collectable biological sample that can replace throat swabs. Oral fluid (OF)—a mixture of saliva and gingival crevicular fluid collected by brushing the gums—has emerged as a promising candidate. It has already been utilized for COVID-19 nucleic acid testing in several countries during the pandemic (3–5). Moreover, given the correlation between antibodies in gingival crevicular fluid and serum, oral fluid can also be applied for antibody detection, and has proven effective for detecting nucleic acids and antibodies for various pathogens (4, 6–9). However, oral fluid is less sensitive than throat swabs, requiring more accurate detection methods. Digital PCR (dPCR), a highly sensitive and precise nucleic acid quantification technology, offers advantages over the routinely used real-time PCR (RT-PCR), including absolute quantification without a standard curve, higher tolerance to inhibitors, and improved sensitivity. dPCR has already been employed in SARS-CoV-2 nucleic acid detection (10–12), suggesting its potential to overcome the limited sensitivity of oral fluid-based assays. Still, the application of dPCR to oral fluid samples has not yet been reported. This study primarily aimed to evaluate the novel combination of dPCR with OF samples for SARS-CoV-2 nucleic acid detection, which has not been previously reported. We compared this novel method against the standard approach of real-time PCR (RT-PCR) on throat swabs. Additionally, we assessed the feasibility of using OF for SARS-CoV-2 antibody detection against serum-based testing. Our findings aim to provide scientific evidence for adopting OF as a non-invasive sampling method enhanced by the superior sensitivity of dPCR.

## 2 Materials and methods

### 2.1 Study population

From 2022 to 2023, 213 confirmed COVID-19 cases were included in Beijing Haidian Hospital and Beijing Ditan Hospital. The inclusion criteria refer to the “COVID-19 Infection Diagnosis and Treatment Program (Trial version 10)” issued by the National Health Commission. Paired oral fluid and throat swabs were collected simultaneously from each individual. Exclusion criteria included: (1) Patients with mismatched samples of throat swabs and oral fluid; (2) The amount of throat swab or oral fluid collected does not meet the requirements; (3) Patients who are allergic to oral fluid materials (few people are allergic).

### 2.2 Study methods

#### 2.2.1 Sample collection and processing

A total of 213 paired throat swab and OF were collected during the study period. A subset of 108 paired samples was randomly selected for dPCR analysis due to budget constraints.

##### 2.2.1.1 Throat swabs and serum sample collection and processing

Throat swab and serum samples were collected according to the protocol for the prevention and control of COVID-19 (Trial Version 8). Throat swabs were preserved using an inactivated sample tube with guanidine salt.

##### 2.2.1.2 Oral fluid sample collection and processing

Oral fluid was self-collected according to the manufacturer’s instructions under the guidance of staff. Briefly, gently wipe the gum line with a foam swab for 90 s until saturated. Place the swab into a collection tube, stored at 4 °C, and transport to the laboratory within 24 h. For processing, add 1 mL of elution buffer to the tube. Press the swab against the inner wall to release the sample, then snap off the handle. After capping the tube, invert it and centrifuge at 250 × g for 1 min. Collect the supernatant into a 2 mL tube and store at −20 °C.

#### 2.2.2 RT-PCR detection of SARS-CoV-2 nucleic acid

##### 2.2.2.1 Nucleic acid extraction

Total nucleic acids were extracted from the samples using automated equipment (Thermo Scientific^™^ KingFisher^™^ Flex Magnetic Particle Processors, Thermo Fisher, MA, United States).

##### 2.2.2.2 Nucleic acid detection

A real-time PCR-based commercial diagnostic kit (The Detection Kits for SARS-CoV-2 nucleic acids, Guangzhou Daan Gene Co., Ltd., Guangzhou, China) was used for nucleic acid detection of SARS-CoV-2. The target genes were the ORF1ab gene and the N gene. The operation was carried out strictly following the kit instructions and instrument manual. If the ORF1ab and N genes show typical S-type amplification curves and the cycle threshold (Ct) values are less than 40, the result is positive; otherwise, it is negative.

#### 2.2.3 dPCR detection of SARS-CoV-2 nucleic acid

The nucleic acids were extracted as described above. According to the instructions of the 2019-nCoV nucleic acid detection kit (fluorescent PCR method) (Xinyi Manufacturing Technology (Beijing) Co., Ltd., Catalog No. 13443), and droplets were generated using the company’s sample preparation apparatus (Drop Maker M1). After a one-step reverse transcription amplification reaction, the quantitative detection was realized by using the biochip analyzer (Chip Reader R1). According to the detection results, the appropriate positive regions of ORF1ab and N genes were delimited, and copies of each detection target were obtained.

#### 2.2.4 IgG antibody detection of SARS-CoV-2

##### 2.2.4.1 Detection of serum antibody

Serum antibodies were detected by the SARS-CoV-2 IgG antibodies detection kit (magnetic particle chemiluminescence method) (Bioscience, Chongqing, China; lot number: G202108003). The results were judged as follows: the S/CO (luminescence value/critical value) ratio ≤1, the result was positive; otherwise, it is negative.

##### 2.2.4.2 Detection of oral fluid antibody

SARS-CoV-2 IgG antibodies in oral fluid were detected by the magnetic particle chemiluminescence method developed by the Beijing Center for Disease Control and Prevention (6). The results were determined by the serum antibody method mentioned above.

### 2.3 Statistical analysis

Continuous variables were presented as mean or median and categorical variables were presented as rates (%). Pearson’s *χ*^2^ or Fisher’s exact test was applied to examine differences between categorical variables. A two-sided *p*-value of less than 0.05 was considered significant. Kappa analysis was used to evaluate the detection agreement between the two sampling methods or detection methods, with scores as follows: >0.75 indicated excellent agreement, 0.60 < kappa ≤ 0.75 indicated high agreement, 0.40 < kappa ≤ 0.60 indicated moderate agreement, and ≤0.40 indicated poor agreement. The thresholds of the coefficient of determination (*R*) are defined as follows: When 0 ≤ ∣*R*∣ < 0.3, it indicates a low-degree linear correlation. When 0.3 ≤ ∣*R*∣ < 0.5, it represents a moderately-low-degree linear correlation. When 0.5 ≤ ∣*R*∣ < 0.8, it shows a moderate-degree linear correlation. When 0.8 ≤ ∣*R*∣ ≤ 1, it signifies a high-degree linear correlation. All statistical analyses were done using SPSS statistical software version 19.0 (SPSS Inc., Chicago, United States). Figures were drawn by GraphPad Prism 8 software.

## 3 Results

### 3.1 The concordance rate of paired samples between RT-PCR and dPCR methods

A total of 213 paired throat swabs and oral fluid samples from confirmed COVID-19 cases were collected. The results of RT-PCR showed that the detection rates of SARS-CoV-2 by oral fluid and throat swab samples was 80.75% (172/213) and 88.73% (189/213), respectively (*χ*^2^ = 54.09, *p* < 0.001). With throat swabs as the reference method, oral fluid had a sensitivity of 87.83% (166/189). The total agreement rate and kappa value were 86.38% and 0.48, indicating that OF’s detection results align well with traditional throat swabs in most scenarios. The different Ct values of the throat swab showed that when the Ct value of the throat swab was ≤25, 92% of the paired oral fluid samples were positive (Table 1). The result specifies the scenarios where OF performs best: in the acute infection phase, OF’s positive detection rate is close to that of throat swabs.

**Table 1:** The coincidence rate between different Ct values of throat swabs and paired oral fluid samples.

Ct values of throat swab	Genes	Positive samples		Coincidence rate (%)
		Throat swab	Paired oral fluid	
Ct ≤ 25	ORF1ab	53	49	92.45
	N	55	51	92.73
25 < Ct ≤ 30	ORF1ab	89	77	86.52
	N	88	75	85.23
30 < Ct < 40	ORF1ab	47	40	85.11
	N	44	37	84.09

Further, 108 paired throat swabs and oral fluid samples were selected for dPCR detection. Among 108 throat swabs, the detection rate of RT-PCR method was 92.59% (100/108), the detection rate of dPCR methods was 95.37% (103/108) (Table 2), and the kappa value between the two methods was 0.59 (*p* < 0.001). For oral fluid samples, RT-PCR method and dPCR detection rate were 85.19% (92/108) and 86.11% (93/108), respectively. The two detection methods had a higher kappa value of 0.74 (*p* < 0.001), indicating stronger replaceability between the two detection methods.

**Table 2:** The detection result of throat swab samples by RT-PCR and dPCR.

Throat swab		RT-PCR		
		Positive	Negative	Total
dPCR	Positive	99	4	103
	Negative	1	4	5
	Total	100	8	108

The detection rates of SARS-CoV-2 from oral fluid samples varied across different age groups. In the <60-year-old group and ≥60-year-old group, the detection rates of the dPCR method were 84.81% (67/79) and 86.20% (25/29), respectively; the detection rates of the RT-PCR method were 83.54% (66/79) and 93.10% (27/29), respectively. The result showed that greater caution should be exercised regarding the OF detection rate in the ≥60-year-old patient group.

For throat swab samples, four specimens that yielded negative results in RT-PCR tests, with single-gene cycle threshold (Ct) values spanning from 26 to 40, demonstrated positive outcomes in dPCR. The viral loads in these four samples, as determined by dPCR, ranged from 1.3 to 4449.5 copies per test. Moreover, all the paired oral fluid samples corresponding to these four throat swab samples were positive (Table 2).

For oral fluid samples, four specimens initially showed negative results in the reverse-transcription polymerase chain reaction (RT-PCR) assay. The single-gene cycle threshold (Ct) values of these samples in the RT-PCR test were in the range of 36–40. However, when subjected to digital polymerase chain reaction (dPCR), these same four samples tested positive. The viral loads measured by dPCR in these four oral fluid samples ranged from 6 to 12.8 copies per test. Additionally, all the paired throat swabs corresponding to these four oral fluid samples were positive, as presented in Table 3.

**Table 3:** The detection result of oral fluid samples by RT-PCR and dPCR.

Oral fluid		RT-PCR		
		Positive	Negative	Total
dPCR	Positive	89	4	93
	Negative	3	12	15
	Total	92	16	108

Thus, dPCR improves the detection of low viral load samples to reduce missed diagnoses, and the complementarity between throat swabs and OF enhances diagnostic reliability.

### 3.2 Comparison of the performance of oral fluid and throat swab nucleic acid detection

**Figure 1:**
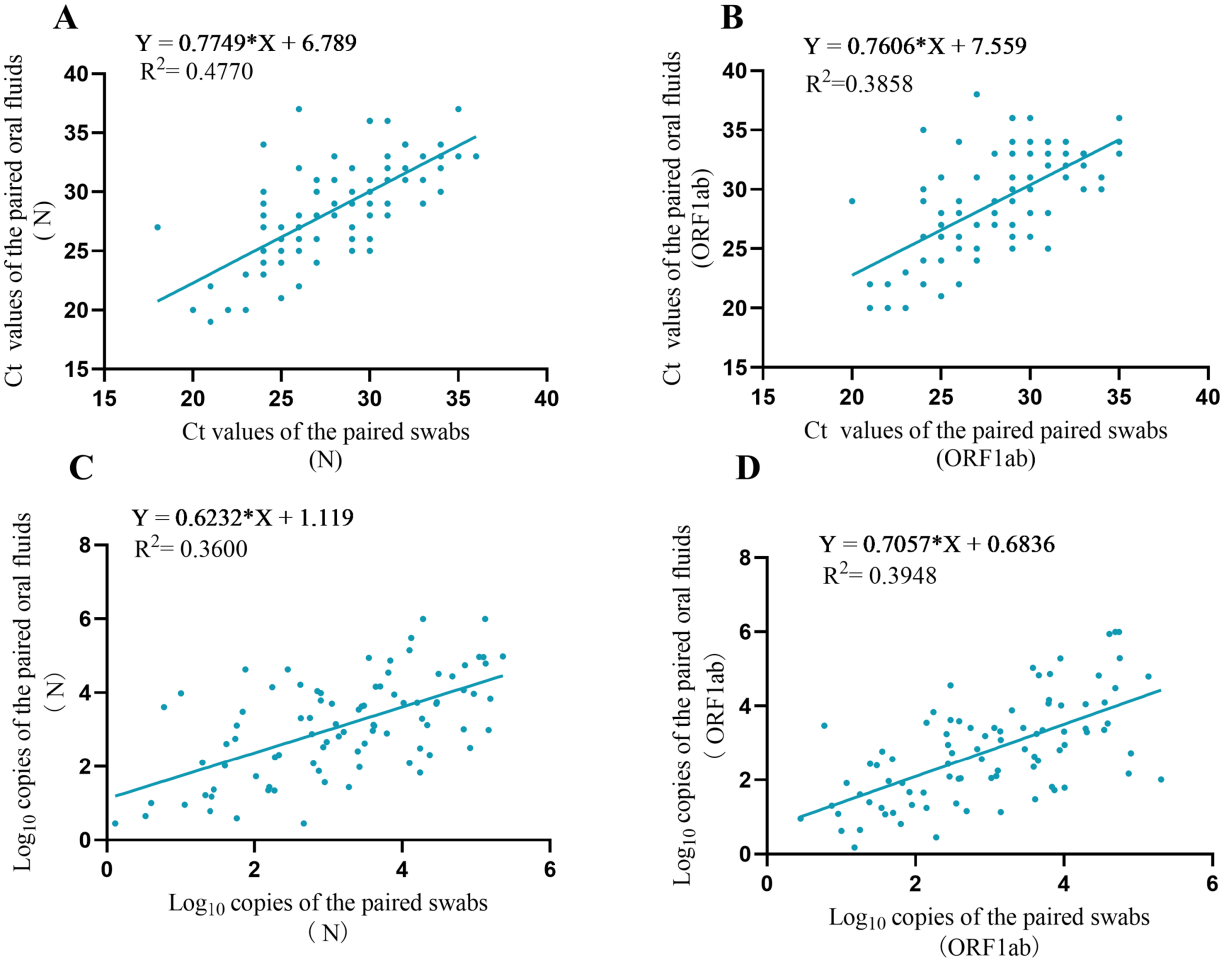
Correlation analysis of different samples detected by two methods. **(A)** Correlation analysis between swabs and paired oral fluid of Ct values for N gene. **(B)** Correlation analysis between swabs and paired oral fluid of Ct values for ORF1ab gene. **(C)** Correlation analysis between swabs and paired oral fluid log_10_ copies for N gene. **(D)** Correlation analysis between swabs and paired oral fluid log_10_ copies for ORF1ab gene.

The correlation analysis of positive oral fluid and throat swab samples showed that there was a moderate linear correlation between the two types of samples by RT-PCR, and the correlation coefficients (*r*) of the N gene and ORF1ab gene were 0.69 and 0.62, respectively. Correlation analysis was performed after log10 was applied to the detection results of dPCR method. The correlation coefficients r of the N gene and ORF1ab gene of the two types of the samples were 0.6 and 0.62, respectively (Figure 1).

### 3.3 Characteristics of viral load of oral fluid and throat swab samples

**Figure 2:**
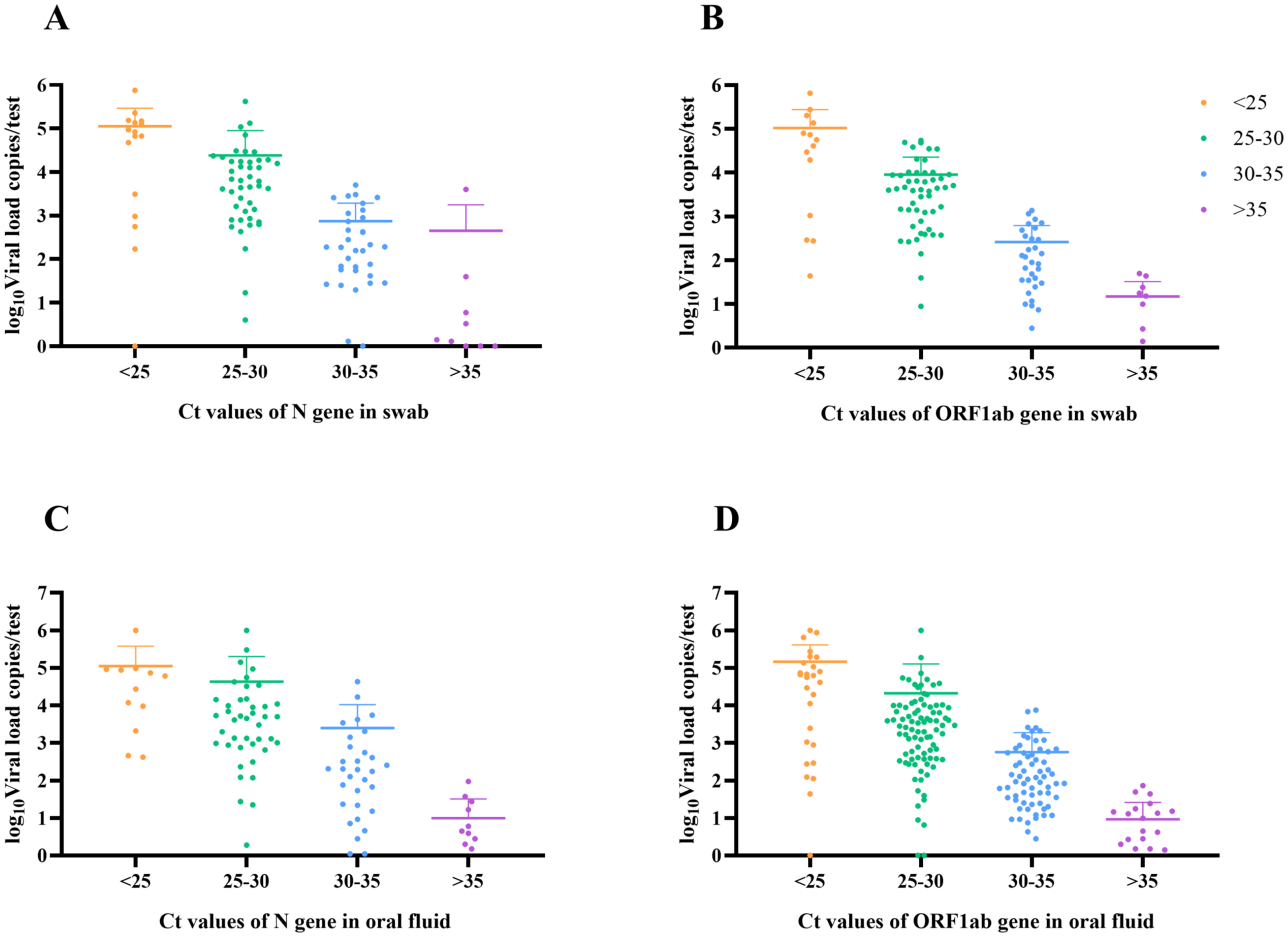
Viral load characteristics in throat swab and oral fluid samples with different Ct values. **(A)** Viral load in throat swab with different Ct values for N gene. **(B)** Viral load in throat swab with different Ct values for ORF1ab gene. **(C)** Viral load in oral fluid with different Ct values for N gene. **(D)** Viral load in oral fluid with different Ct values for ORF1ab gene.

Among the 108 samples detected by dPCR, the viral load of throat swabs ranged from 0 to 7.6 × 10^5^ copies/test, and the viral load of oral fluid samples ranged from 0–8.7 × 10^5^ copies/test. The viral load of both samples decreased gradually with the increase in Ct value. For throat swab and oral fluid samples with N gene Ct values ≥35, the ranges of viral loads were from 3982.1 copies/test down to 0 and from 94.7 copies/test down to 0, respectively (Figure 2).

In the same case, 38.89% (N gene) −41.67% (ORF1ab gene) of oral fluid samples had higher viral loads than throat swab samples, suggesting that the viral loads in the two types of samples were slightly different. Further analysis of viral loads in different age groups showed that there was no difference in viral loads between oral fluid and throat swab samples in the <60-year-old age group (N gene: *p* = 0.126; ORF1ab gene: *p* = 0.34). In the ≥ 60-year-old age group, there was no difference in viral loads between oral fluid and throat swab samples for the N gene (*p* = 0.121), but there was a statistically significant difference in the ORF1ab gene (*p* = 0.038).

**Figure 3:**
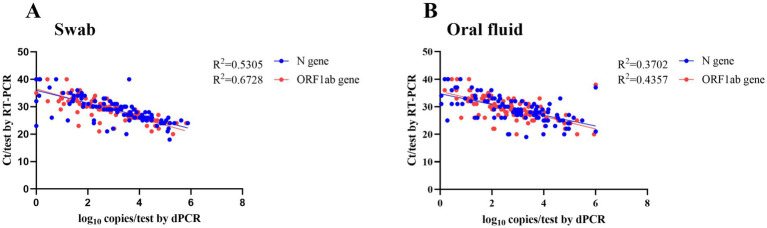
The linear relationship between Ct values and log_2_ copies/test. **(A)** The linear relationship between Ct values and viral load in throat swab. **(B)** The linear relationship between Ct values and viral load in oral fluid.

As shown in Figure 3, there is a high correlation between the Ct values detected by the RT-PCR method and the log_10_ copies/test detected by dPCR in two types of the samples (throat swab: ORF1ab, *R* = 0.82; N, *R* = 0.73; oral fluid: ORF1ab, *R* = 0.66; N, *R* = 0.61), indicating that the two methods have a great agreement. Among the RT-PCR negative samples, four cases were positive by dPCR, with viral loads ranging from 3982.1 copies/test to 1.3 copies/test; of the samples that were negative for oral fluid by RT-PCR, three cases were positive by dPCR, with viral loads ranging from 6 to 2.8 copies/test. Compared with oral fluid (OF) samples, the viral load in throat swabs was approximately 1.3–1.7 times higher, indicating that a more sensitive detection method would be more suitable for OF samples.

### 3.4 Comparison of antibody detection between oral fluid and serum samples

**Figure 4:**
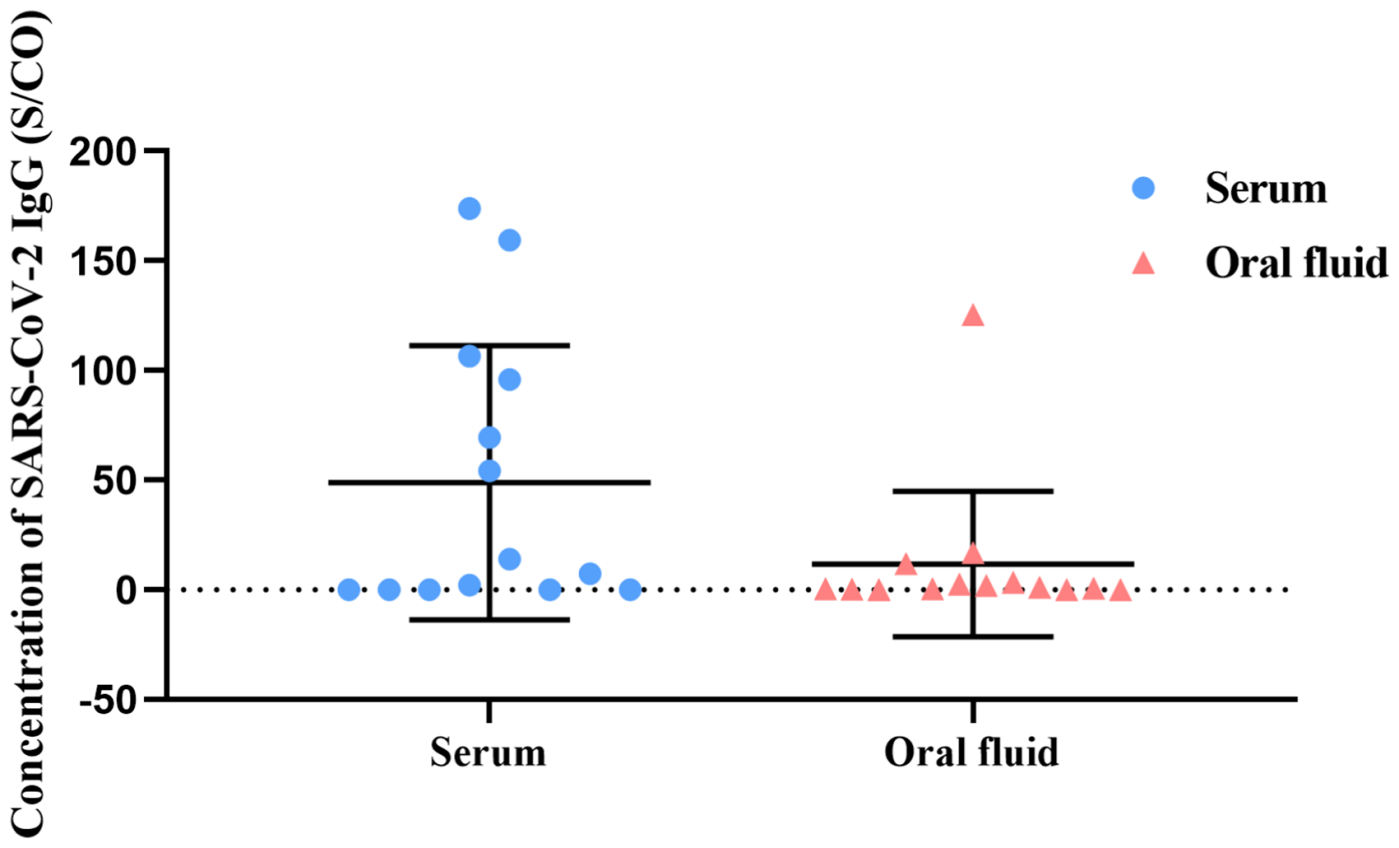
Comparison of SARS-CoV-2 IgG antibodies in serum and oral fluid of COVID-19 cases.

Due to poor compliance and the difficulty in collecting blood, a total of 14 serum samples from confirmed COVID-19 cases were collected. The SARS-CoV-2 antibodies in serum and oral fluid samples were detected by chemiluminescence, and the results were shown in Figure 4. The IgG antibody detection rates in serum and oral fluid samples were 64.28% (9/14) and 50% (7/14), respectively. The consistency of detection results between the two sample types was 71.42% (10/14). The results highlighted OF’s potential for long-term immune monitoring.

## 4 Discussion

Oral fluid has been successfully used for the detection and surveillance of pathogens worldwide, such as measles, rubella, mumps, and influenza (7, 13, 14). During the COVID-19 pandemic, saliva was widely used in many countries for nucleic acid detection because of its convenience, showing great consistency with traditional biological samples such as throat swabs. Also oral fluid has been applied for antibody detection against many pathogens including SARS-CoV-2 (13, 15, 16). Though the advantages of safety, convenience, and non-invasiveness, the improvement of sensitivity and specificity was also needed.

In this study, the detection rate of oral fluid nucleic acid (80.75%, 172/213) was lower than that in To et al. (17) (91.71%, 11/12), and higher than that in Fang et al. (18) (78.1%, 25/32). The observed heterogeneity can be primarily attributed to sample size, as well as variations in reagent sensitivity and specificity, which may introduce technical bias. Notably, oral fluid consistently demonstrated marginally lower detection rates compared to throat swabs, with self-collection methodology identified as a critical confounder. Insufficient mucosal contact, incomplete collection volumes, or contamination from oral commensals may reduce viral load in the specimen, particularly for pathogens preferentially shed in the lower respiratory tract. Previous studies have shown that self-collected samples are associated with higher rates of inadequate specimens compared to clinician-collected swabs (19). However, the two sample types were in good agreement in our study, consistent with previous studies by Huang et al. (6, 20). Meanwhile, for samples with a Ct <40, the consistency between oral fluid and throat swab samples exceeded 84%, improving with lower Ct values, indicating the use of oral fluid as a reliable alternative in high viral load scenarios and in challenging settings such as severe cases, pediatric patients, and large-scale screening (21).

dPCR has been applied to the detection of SARS-CoV-2 (11) due to its high sensitivity and specificity, especially in RT-PCR negative suspected cases (10). Our study showed high consistency between RT-PCR and dPCR for both oral fluid and throat swabs, as well as a higher agreement between the two methods for oral fluid (kappa = 0.74). A linear correlation between RT-PCR Ct values and the dPCR copy numbers was observed, suggesting the reliability of the two methods at high viral load (12). With a reported detection threshold below 10 copies (22), dPCR offers a clear advantage for low viral load samples such as oral fluid. dPCR detected positives in 4 throat swab samples and 3 oral fluid samples that were negative by RT-PCR (10, 11), confirming its superior sensitivity near the detection limit (23). These findings suggest dPCR can serve as a valuable supplementary method to reduce false negatives in RT-PCR.

Oral fluid has been widely used in serum antibody detection. In our study, the consistency of SARS-CoV-2 IgG antibody between oral fluid and serum was 71.42%, which was lower than the previous study results (95.06–100%) (6, 24). This discrepancy may be due to the small sample size. Expanding the sample size in future studies will help clarify the utility of oral fluid in serological monitoring.

A key advantage of oral fluid lies in its biosafety profile. It allows self-collection at home, minimizing cross-infection risks during mass sampling and reducing exposure for healthcare workers. Collection tubes do not require viral transport media, eliminating leakage risks during transport. Processing is also simplified: after adding elution buffer and centrifugation, the supernatant can be safely stored, greatly reducing handling risks.

## 5 Conclusion

In conclusion, our findings support oral fluid as a valuable biological sample for SARS-CoV-2 detection, offering distinct advantages of being non-invasive, safe, convenient, and efficient for large-scale use. The application of highly sensitive methods like dPCR can effectively compensate for its inherent limitations of lower viral loads and antibody titers, thereby enhancing detection reliability. Therefore, oral fluid can serve as a practical alternative to throat swabs for nucleic acid detection and as a supplementary tool to serum for antibody detection, demonstrating broad application potential in the monitoring and diagnosis of infectious diseases.

## Data Availability

The raw data supporting the conclusions of this article will be made available by the authors, without undue reservation.
